# Outpatient and Inpatient Service Use by Chinese Adults Living in Rural Low-Income Households

**DOI:** 10.1093/geroni/igaa057.263

**Published:** 2020-12-16

**Authors:** Peiyi Lu, Jun Yao, Chunyu Yang, Mack Shelley, Li Zhang

**Affiliations:** 1 Iowa State University, Ames, Iowa, United States; 2 NANJING MEDICAL UNIVERSITY, NANJING, Jiangsu, China; 3 Nanjing University of Information Science and Technology, NANJING, Jiangsu, China; 4 Nanjing University of Chinese Medicine, NANJING, Jiangsu, China

## Abstract

Background: This study applied the Andersen Model of Health Care Utilization to explore the variables associated with health service use among Chinese adults living in rural low-income households. Method: A survey of 2,429 adults living in 787 low-income households in Jiangsu, China was conducted. Respondents were asked the presence of outpatient service in the past one month and the amount of hospitalization in the past one year. Mixed effect logistic and negative binomial models were used to examine the relationship of individual-level and household-level characteristics with health service use. Results: Health condition was the predominant determinant of both outpatient and inpatient service use (Odds Ratio [OR] >1, p<0.001). Individuals living in a poor household were less likely to use outpatient service (OR=0.05, 95% confidence interval CI: 0.00, 0.71), and the longer in poverty status the less likely to use outpatient service (OR=0.92, CI: 0.86, 0.99). Age was associated with lower likelihood to use outpatient service (OR=0.93, CI: 0.93, 1.00), and this relationship was stronger for larger households (OR=1.01, CI: 1.00, 1.01). For inpatient service use, most household-level measures were insignificant. Conclusion: Rural Chinese health service use was influenced primarily by needs variables. Outpatient service use was constrained by household enabling variables. Older adults were at a disadvantage for using outpatient service when the family prioritized younger members in allocating resources. These results suggest the need for policy advocacy to expand insurance reimbursement and improve benefits for poor older adults.

